# Ocular syphilis with optic disc neovascularization treated with bevacizumab evaluated by OCT angiography and electroretinography

**DOI:** 10.1186/s12348-020-00219-x

**Published:** 2020-10-30

**Authors:** Gen Miura, Takayuki Baba, Marie Takeishi, Tomoaki Tatsumi, Hirotaka Yokouchi, Shuichi Yamamoto

**Affiliations:** Department of Ophthalmology and Visual Science, Chiba University Graduate School of Medicine, Inohana 1-8-1, Chuo-ku, Chiba, 260-8670 Japan

**Keywords:** Bevacizumab, ERG, OCTA, Optic disc neovascularization, Syphilis

## Abstract

We present our findings in an atypical case of ocular syphilis with optic disc neovascularization that was treated with intravitreal bevacizumab and followed by multimodal imaging and electroretinography. A 29-year-old man presented with a chief complaint of night blindness of one-year duration. Our initial examination showed that an optic disc neovascularization was present, and the optical coherence tomographic (OCT) images showed a reduction in the length of the ellipsoid zone of both eyes. Fluorescein angiography showed leakage from the optic disc neovascular tissue, and the presence of nonperfused areas in the peripheral retina. Blood test was strongly positive for syphilis. He was administered oral amoxicillin and prednisolone. He was also treated with an intravitreal injection of bevacizumab which led to a rapid suppression of the neovascularization. However, panretinal photocoagulation had to be performed because OCT angiography and fluorescein angiography detected residual neovascularization. Although these treatments suppressed the activity of the ocular syphilis, electrophysiological improvements were not seen even 1 year after the initial treatment. OCT angiography and electroretinogram are useful techniques for monitoring the effectiveness of the treatments.

## Case report

**Fig. 1:**
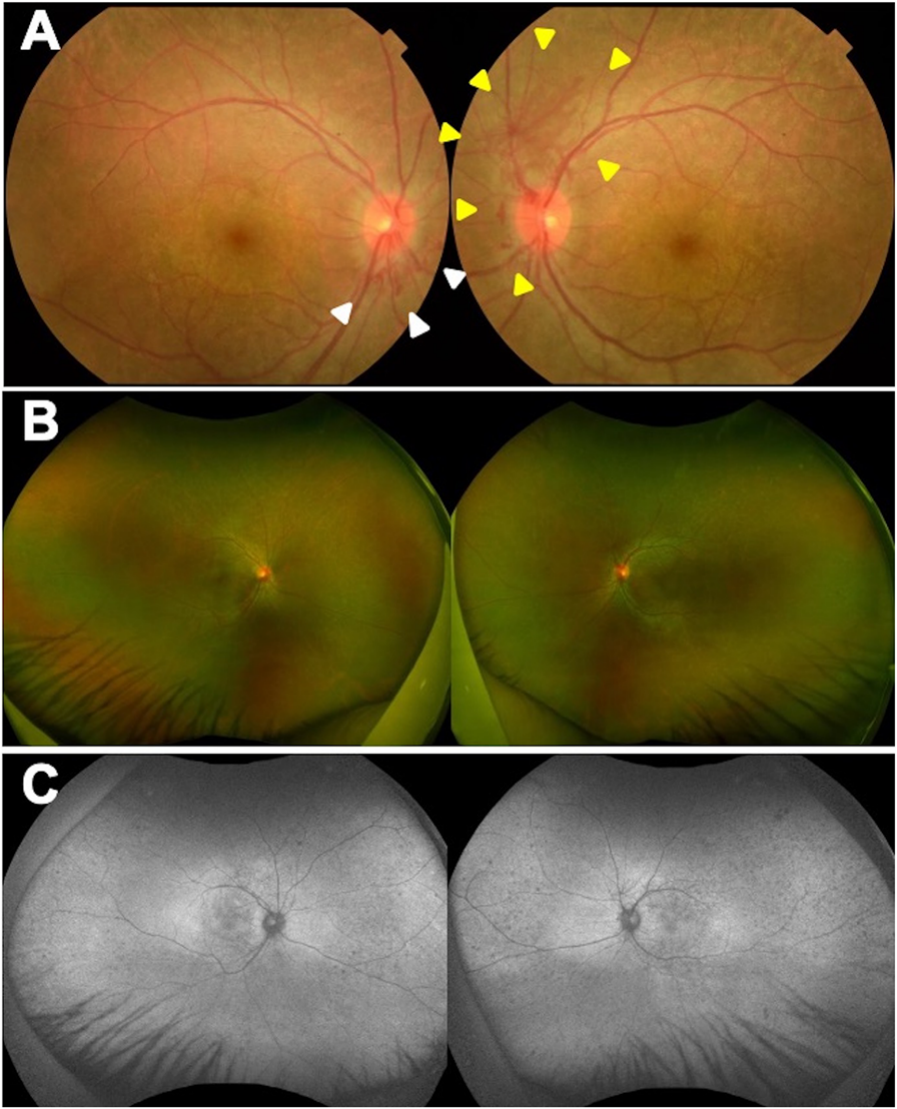
Ocular findings in a patient with syphilis with optic disc neovascularization in both eyes. **a:** Color fundus photograph of both eyes at the initial visit. Bilateral optic disc neovascularization can be seen (white arrowhead of right eye and yellow arrowhead of left eye). **b:** Ultra-widefield fundus photograph of both eyes at the initial visit. **c:** Ultra-widefield fundus autofluorescence (FAF) of both eyes at the initial visit. Hypo-autofluorescence suggesting retinal pigment epithelium (RPE) atrophy that match retinal white spotted lesions can be seen

**Fig. 2:**
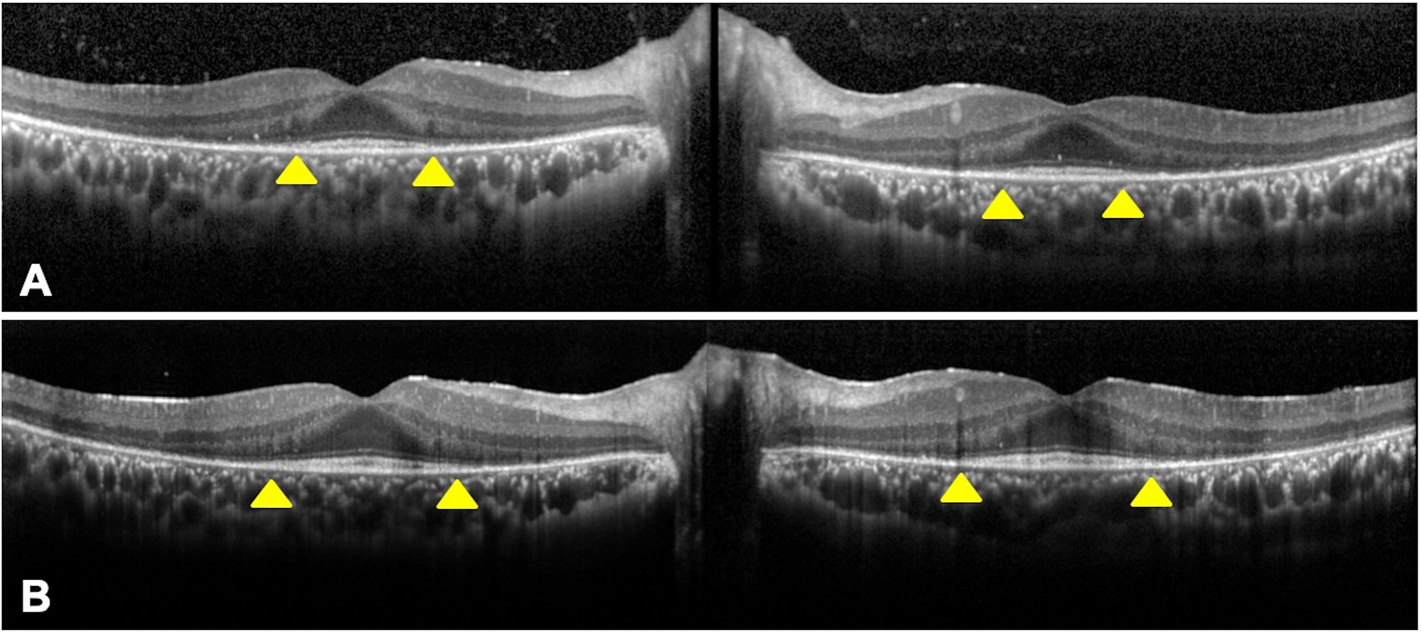
Optical coherence tomographic (OCT) images of both eyes. **a:** OCT at the initial visit. A shortening of ellipsoid zone (EZ) length (yellow arrowhead), slight nodular elevation of foveal EZ, thinning of the outer nuclear layer and punctate hyperreflectivity of the choroid can be seen. **b:** OCT at 1 year after the initial visit. EZ length was slightly prolonged (yellow arrowhead) and nodular elevations of the foveal EZ has decreased

**Fig. 3:**
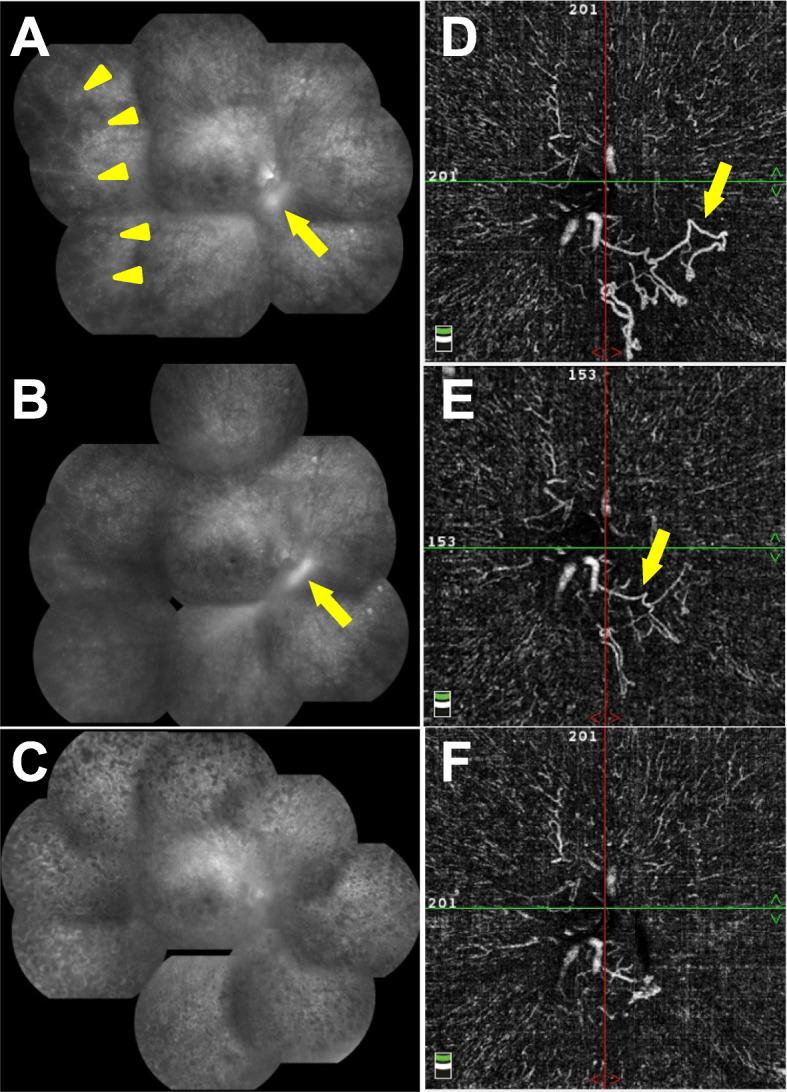
Fluorescein angiography (FA) and OCT angiography (OCTA) images of the right eye. **a:** FA at the initial visit. Leakage from optic disc neovascular tissue (yellow arrow), and nonperfused areas in the peripheral retina (yellow arrowhead) and window defects can be seen. **b:** FA after Intravitreal injection of bevacizumab (IVB). Leakage from residual neovascularization (yellow arrow) can be seen. **c:** FA after panretinal photocoagulation (PRP). **d:** OCTA image of the vitreous layer at the initial visit. Obvious neovascularization (yellow arrow) can be seen. **e:** OCTA image of the vitreous layer after IVB. Residual neovascularization (yellow arrow) can be seen. **f:** OCTA image of the vitreous layer after PRP

**Fig. 4:**
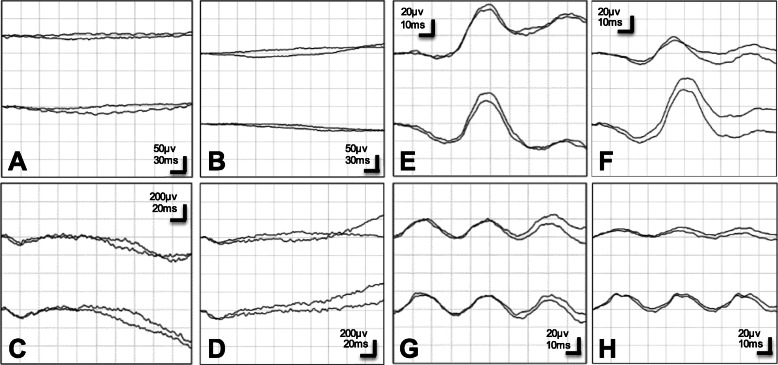
Electroretinogram (ERG) waveforms of both eyes. The upper line shows the ERGs of the right eye and the lower line shows the ERGs of the left eye. The ERGs show no obvious improvement. **a:** Dark-adapted (DA) 0.01 at the initial visit. ERGs were non-recordable. **b:** DA 0.01 at 1 year after the initial visit. **c:** DA 3.0 at the initial visit. **d:** DA 3.0 at 1 year after the initial visit. **e:** Light adopted (LA) 3.0 at the initial visit. **f:** LA 3.0 at 1 year after the initial visit. **g:** LA 30 Hz flicker at the initial visit. **h:** LA 30 Hz flicker at 1 year after the initial visit

A 29-year-old man reported that he had night blindness in both eyes for about 1 year. He had no family history of retinal disease. At the initial examination, his visual acuity was 20/20 in both eyes, and the intraocular pressure was 15 mmHg in right eye and 13 mmHg in left eye. Slit-lamp examination did not show cells or flare in the anterior chamber, rubeosis iridis, or vitreous opacities. There were no obvious inflammatory cells in the vitreous. Ophthalmoscopy showed neovascularization on both optic discs (Fig. 1a). White spotted lesions were found throughout the retina, however no pigmentary deposits or attenuation of the retinal vessels was detected (Fig. 1b). Fundus autofluorescence (FAF) showed hypo-autofluorescence suggesting atrophy of the retinal pigment epithelium (RPE), and the sites of the hypo-autofluorescence atrophy matched the retinal white-spotted lesions (Fig. 1c). Optical coherence tomography (OCT) showed a shortening of the length of the ellipsoid zone (EZ), slight nodular elevation of the foveal EZ, thinning of the outer nuclear layer and punctate hyperreflectivity of the choroid (Fig. 2a, b). Fluorescein angiography (FA) showed leakage from the neovascular tissue, nonperfused areas in the peripheral retina, and window defects indicating the sites of the RPE atrophy (Fig. 3a, b). Electroretinography (ERG) showed that the dark-adapted (DA) 0.01 ERGs were non-recordable from both eyes (Fig. 4a). The amplitudes of the DA 3.0 ERGs were markedly reduced (Fig. 4c). The amplitudes of both the light-adapted (LA) 3.0 1 Hz and 30 Hz ERGs were also reduced (Fig. 4e, g), but were relatively better preserved than the DA ERGs.

No abnormalities were found on the chest X-rays and ultrasound evaluations of the carotid artery. Cutaneous eruptions were not observed. Systemic workup including antinuclear antibodies, antidouble-stranded DNA, antineutrophilic cytoplasmic antibodies, rheumatoid factor, IgG4, angiotensin-converting enzyme serum lysozyme, T-SPOT, Mycoplasma pneumoniae antibody, toxoplasmosis IgG and IgM, Epstein-Barr virus-viral capsid antigen IgG and IgM, IgG and IgM of varicella-zoster virus and herpes simplex virus, angiotensin converting enzyme, lysozyme, myeroperoxidase and proteinase3 ANCA, antiSS-A/Ro, antiSS-B/La, antiU1-ribonucleoprotein antibody, and soluble interleukin-2 receptor were all unremarkable. The patient had a mildly elevated C-reactive protein.

The serological test also showed an elevated rapid plasma reagin value to 512 RU (normal value: < 1 RU), and a *T. pallidum* hemagglutination value of 40,960 TU (normal value: < 80 TU). He was diagnosed with syphilis. He tested negative for HIV, gonorrhea, and chlamydia. After the diagnosis was confirmed, he was treated with 1500 mg/day of oral amoxicillin for 10 months and with 30 mg/day of oral prednisolone for 6 weeks.

Intravitreal injection of bevacizumab (IVB) were performed on both eyes with the start of oral treatment, and the optic disc neovascularity quickly regressed ophthalmoscopically. Because residual neovascularity was confirmed by OCT angiography (OCTA) and FA, panretinal photocoagulation (PRP) was performed. A small number of vitreous hemorrhages and mild macular edema were observed during the recovery period, however both abnormalities were resolved and no additional treatment was given.

One year after the initial treatment, the visual acuity was maintained at 20/20, and FA demonstrated that the ischemic changes were resolved (Fig. 3c).

OCT revealed that length of EZ was slightly prolonged, and the nodular elevations of foveal EZ were improved (Fig. 2).

OCTA showed a regression of the optic disc neovascularization after the IVB and further regression after the PRP (Fig. 3d-f). No significant changes were seen in the FAF images (Fig. 1c). The ERG waveforms showed no obvious improvement which indicated that the retinal damage was probably permanent (Fig. 4).

## Discussion

Ocular syphilis presents with different and nonspecific signs and symptoms. For the eye, iridocyclitis, intermediate and posterior uveitis, scleritis, keratitis, chorioretinitis, retinal vasculitis and neuritis have been reported in cases of ocular syphilis [1, 2]. In our case, no obvious signs of inflammation were observed in the anterior segment or intermediate optic media. No abnormal color tone or edema was detected in the optic nerve head, and leakage from the optic disc except from the neovascular tissue, was not significant in the FA images. In addition, there were no obvious systemic signs of syphilis. This case was characterized by the bilateral optic disc neovascularization, retinal ischemia, and extensive retinal pigment epithelium atrophy of both eyes. These clinical findings indicated that this was a case of atypical ocular syphilis. The presence of nonperfused areas in the peripheral retina in FA suggested that vasculitis or vascular occlusion occurred not only around the optic disc where the neovascular tissue was present but also throughout the retina which resulted in the ischemic changes.

A case of ocular syphilis complicated by retinal vasculitis, proliferative retinopathy, and vitreous hemorrhages was reported by Kobayashi et al. [3] Similar to our case were the severe retinal ischemic changes and lack of systemic findings. However, their case differed from our case by the presence of obstructive retinal vasculitis. In addition, the visual acuity in our case was good throughout the course of the disease. Also, our case had window defects throughout the retina which was not reported for their cases. The speed of progression of the two eyes was different in their case which was not true in our case. The findings indicated the necessity to perform appropriate treatments promptly to prevent vitreous hemorrhage.

There are other cases of ocular syphilis that required vitreoretinal surgery. In one case, vitreous hemorrhage developed from occlusive retinal vasculitis [4]. Other cases developed rhegmatogenous and tractional retinal detachment that progressed to retinal necrosis [5, 6]. Haug et al. reported that four of 11 acute syphilitic panuveitis eyes (36.4%) had a redetachment of the retina. These reports suggest that it is important to prevent progression to vitreous hemorrhage and proliferative changes. Therefore, a rapid suppression of the ischemic changes using IVB prior to photocoagulation therapy may be effective in cases with severe neovascularizations.

It is difficult to detect inflammation by OCTA unlike FA, however OCTA can detect nonperfused and neovascular areas. OCTA is occasionally better in detecting vascular abnormalities, retinal capillary changes, and neovascular formation than FA. Abucham-Neto et al. studied vasculitis cases with syphilis and reported that early peripapillary neovascular proliferation, telangiectasia, and neovascularization obscured by retinal hemorrhage were detected better by OCTA than by FA [7]. Residuals of neovascularization around the optic disc after IVB were clearly confirmed by OCTA in our case. Therefore, OCTA can be useful complementary diagnostic tool in cases of uveitis especially in cases with ischemic changes and neovascularization as our case. Because there are few reports of OCTA regarding syphilis, further studies about OCTA of ocular syphilis with larger sample sizes are needed.

Electroretinographic studies have not been performed on cases of ocular syphilis that underwent PRP with severe ischemia such as in our case. However, there are several reports of ERG evaluations on comparatively mild ocular syphilis cases. Many of the reports showed that treatment for syphilis improved the ERG waveforms [8, 9]. No obvious ERG improvements were observed in our case unlike the earlier reports. It has been reported that PRP treatments reduced the ERG amplitudes significantly [10]. Therefore, the ERG amplitudes decrease due to PRP treatment in addition to the prolongation of retinal disfunction due to ocular syphilis may have occurred in our case. The ERG evaluations of cases treated with PRP should be performed carefully.

A limitation of this case report is that magnetic resonance imaging of the brain and spinal cord and lumbar puncture were not performed to further evaluate the evidence of neurosyphilis.

In summary, we have presented our findings in an atypical case of ocular syphilis with optic disc neovascularization. The neovascularization on the optic disc was suppressed by IVB, however its effect was transient and PRP treatment was required. In addition to FA, OCT angiography was useful for evaluating the optic disc neovascular activity. ERG evaluations were also important because ERG disorders may be prolonged as in this case even after the activity of ocular syphilis is suppressed by systemic and focal treatments.

## Data Availability

Not applicable to this case report.

## Abbreviations

OCT: Optical coherence tomography
FAF: Fundus autofluorescence
RPE: Retinal pigment epithelium
EZ: Ellipsoid zone
FA: Fluorescein angiography
ERG: Electroretinography
DA: Dark-adapted
LA: Light-adapted
IVB: Intravitreal injection of bevacizumab
OCTA: OCT angiography
PRP: Panretinal photocoagulation
